# Prognostic values of distinct CBX family members in breast cancer

**DOI:** 10.18632/oncotarget.21325

**Published:** 2017-09-28

**Authors:** Yuan-Ke Liang, Hao-Yu Lin, Chun-Fa Chen, De Zeng

**Affiliations:** ^1^ The Breast Center, Cancer Hospital of Shantou University Medical College, Shantou, China; ^2^ Department of Medical Oncology, University Medical Center Groningen, University of Groningen, Groningen, The Netherlands; ^3^ Department of Breast and Thyroid Surgery, The First Affiliated Hospital of Shantou University Medical College, Shantou, China; ^4^ Department of Medical Oncology, Cancer Hospital of Shantou University Medical College, Shantou, China

**Keywords:** CBX, breast cancer, prognostic values, tamoxifen, chemosensitivity

## Abstract

Chromobox (CBX) family proteins are canonical components in polycomb repressive complexes 1 (PRC1), with epigenetic regulatory function and transcriptionally repressing target genes via chromatin modification. A plethora of studies have highlighted the function specifications among CBX family members in various cancer, including lung cancer, colon cancer and breast cancer. Nevertheless, the functions and prognostic roles of distinct CBX family members in breast cancer (BC) remain elusive. In this study, we reported the prognostic values of CBX family members in patients with BC through analysis of a series of databases, including *CCLE*, *ONCOMINE*, *Xena* Public Data Hubs, and Kaplan-Meier plotter. It was found that the mRNA expression of CBX family members were noticeably higher in BC than normal counterparts. CBX2 was highly expressed in Basal-like and HER-2 subtypes, while CBX4 and CBX7 expressions were enriched in Luminal A and Luminal B subtypes of BC. Survival analysis revealed that CBX1, CBX2 and CBX3 mRNA high expression was correlated to worsen relapse-free survival (RFS) for all BC patients, while CBX4, CBX5, CBX6 and CBX7 high expression was correlated to better RFS in this setting. Noteworthily, CBX1 and CBX2 were associated with chemoresistance whereas CBX7 was associated with tamoxifen sensitivity, as well as chemosensitivity in breast tumors. Therefore, we propose that CBX1, CBX2 and CBX7 are potential targets for BC treatment. The results might be beneficial for better understanding the complexity and heterogeneity in the molecular underpinning of BC, and to develop tools to more accurately predict the prognosis of patients with BC.

## INTRODUCTION

In addition to gene mutation or dysregulated amplification, abnormality in epigenetic regulation has been recognized to play a prominent role in the tumorigenesis and development of a broad range of cancer types, including lung, colorectal and breast cancer [1–6]. Chromobox (CBX) family proteins are canonical components in polycomb repressive complexes 1 (PRC1), which are epigenetic regulatory complexes that conduct transcriptional repression of target genes via modifying the chromatin [7].

Up to now, eight CBX proteins in human genome have been identified, with similar chemical structure that contains a single N-terminal chromodomain [8]. The CBX proteins are all involved in the regulation of heterochromatin, gene expression, and developmental programs. CBX proteins are further divided into two groups: (1) CBX1, CBX3, and CBX5, also known as heterochromatin protein 1β (HP1β), HP1γ, and HP1α, respectively; (2) CBX2, CBX4, CBX6, CBX7, and CBX8, all having a C-terminal polycomb repressor box, serve as canonical components in PRC1 [8]. However, studies showed that different polycomb group CBX family proteins were associate with distinct regions of chromatin using nonhomologous protein sequences, which imply a specific target of individual polycomb group protein [9, 10].

Increasing evidences have indicated that CBX proteins play pivotal roles in tumor initiation, progression, and development by blocking differentiation and promoting self-renewal of cancer stem cells [11]. Deregulation of CBX proteins in various cancers has been reported [12–17]. Morey and colleagues have suggested that there was non-overlapping function of the polycomb group CBX family of proteins in embryonic stem cells [18]. Recently, Rong-gang MA and colleagues have reviewed the emerging role of CBX proteins in a number of physiological and pathological conditions, suggesting deregulation of CBX proteins were associated with many cancer types [7]. Study by Jie Li and colleagues indicated that CBX4 was a critical regulator of tumor angiogenesis by governing HIF-1a protein [19]. Kanako Shinjo's findings demonstrated that expression CBX7 was associated with poor prognosis in ovarian clear cell adenocarcinoma via TRAIL-induced apoptotic pathway regulation [20]. However, the roles of distinct CBX members in contribution to tumorigenesis and development of BC are largely unknown.

In the current study, we extended the research field to BC based on a variety of large databases, with purpose of determining the prognostic values of distinct CBX family members in BC.

## RESULTS

### CBX family members were distinctively overexpressed in breast cancer

**Figure 1:**
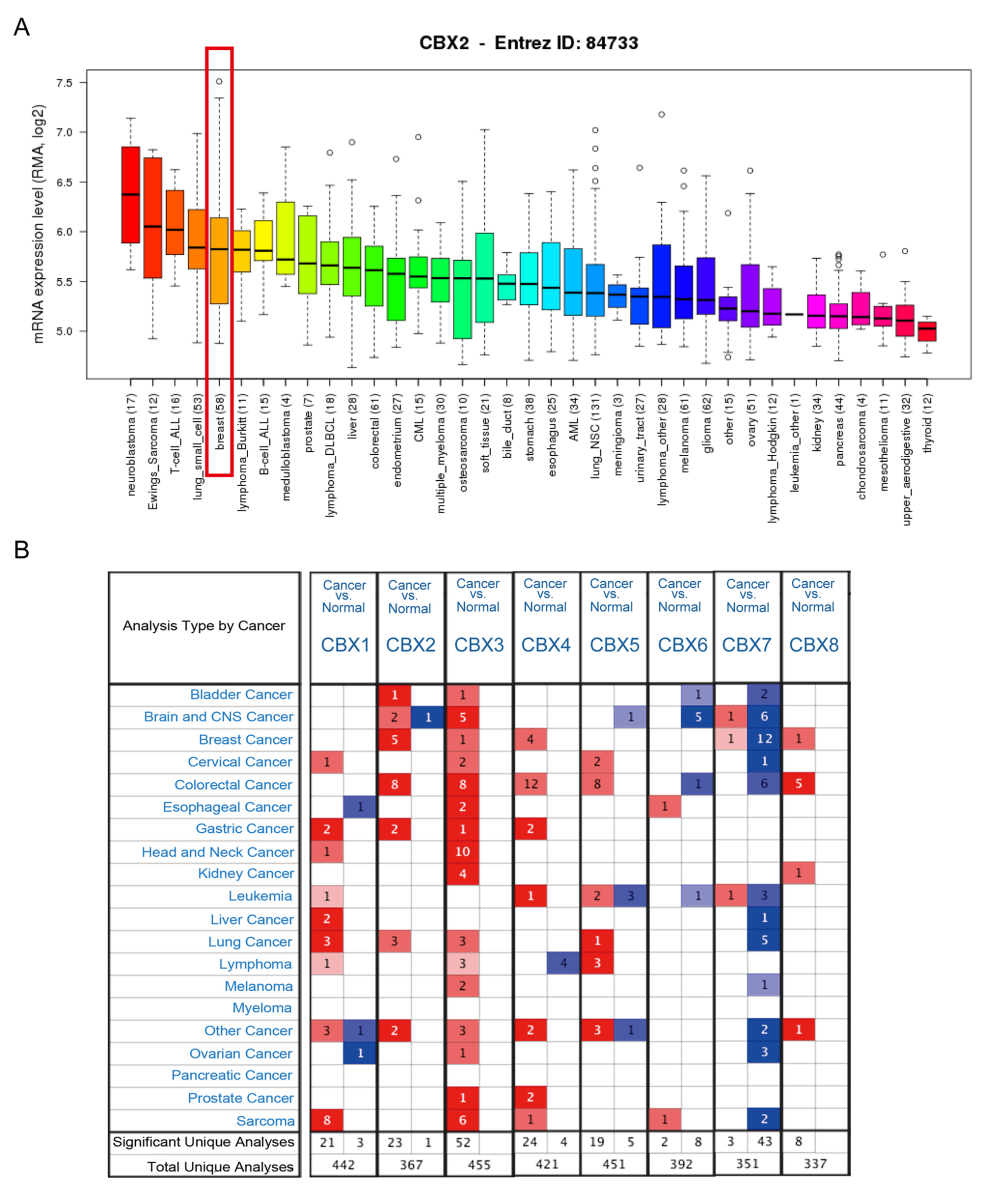
The mRNA expression of CBX family members in different cancer types **(A)** The mRNA expression level of CBX2 analyzed from *CCLE* database. CBX2 ranked the fifth highest in a variety of cancer cell line (shown in red frame). **(B)** CBX family expressions (cancer *vs*. normal tissue) analyzed with *ONCOMINE* database. The graphic demonstrated the numbers of datasets with statistically significant mRNA over-expression (red) or down-expression (blue) of the target gene. The *P* value threshold is 0.01. The number in each cell represents the number of analyses that meet the threshold within those analysis and cancer types. The gene rank was analyzed by percentile of target gene in the top of all genes measured in each research. Cell color is determined by the best gene rank percentile for the analyses within the cell.

Hitherto, 8 CBX family members have been identified in human cancers. *CCLE* analysis demonstrated that mRNA expression level of CBX2 in breast cancer listed the fifth highest among all cancer types (Figure 1A). Also, other CBX family members like CBX3, CBX4, CBX5, CBX6, CBX7 and CBX8 express different patterns in breast cancer compared with other cancer types cells (Supplementary Figure 1, 2).

*ONCOMINE* analysis revealed that CBX2, CBX3, CBX4, CBX7 and CBX8 mRNA expression was significantly higher in BC than normal samples (Figure 1B). CBX2 transcripts were 9.378-fold elevated in breast cancer samples as compared with normal tissues in a dataset with 593 samples that derived from TCGA database (Supplementary Figure 3A).

CBX3 was 2.498-fold elevated in breast cancer samples as compared with normal tissues (*p=*8.88E-9) (Supplementary Figure 3B). CBX4 was 2.604-fold elevated in breast cancer samples as compared with normal tissues (*p=*1.19E-24) (Supplementary Figure 3C). CBX7 was 8.782-fold elevated in breast cancer samples as compared with normal tissues (*p=*1.83E-13) (Supplementary Figure 3D). CBX8 was 2.048-fold elevated in breast cancer samples as compared with normal tissues (*p=*1.10E-16) (Supplementary Figure 3E).

### CBX2 was associated with Basal-like and HER-2 subtypes, while CBX4 and CBX7 was correlated to Luminal A and Luminal B subtypes of breast cancer

**Figure 2:**
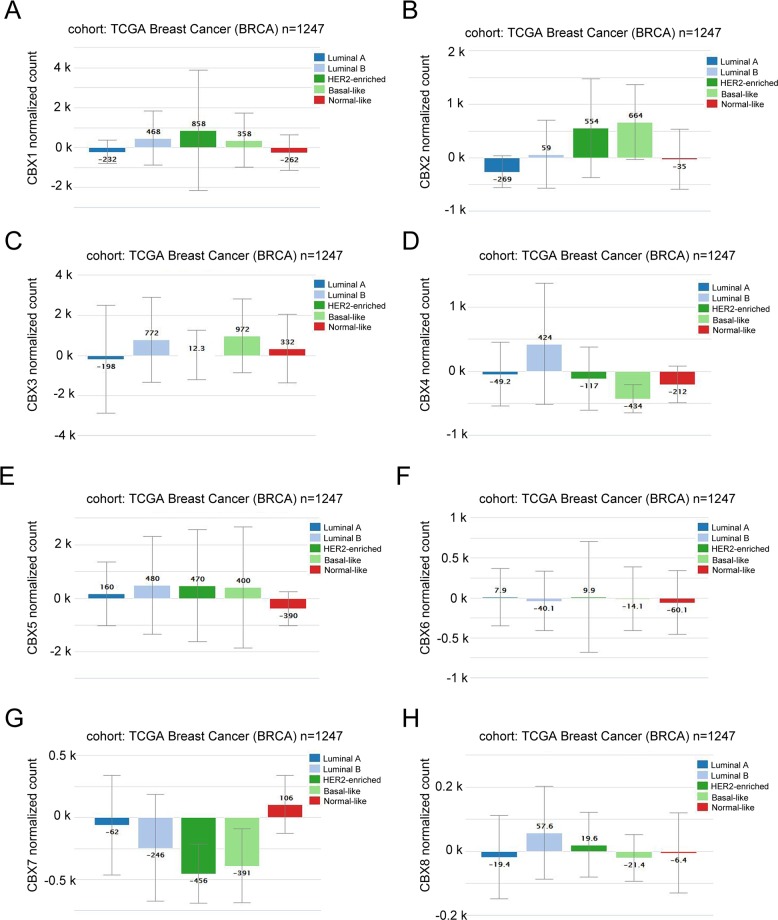
CBX members were distinctively high expressed in breast cancer subtype from *Xena* analysis **(A)** The mRNA expression level of CBX1 in a variety of BC subtype. **(B)** The mRNA expression level of CBX2 in a variety of BC subtype. **(C)** The mRNA expression level of CBX3 in a variety of BC subtype. **(D)** The mRNA expression level of CBX4 in a variety of BC subtype. **(E)** The mRNA expression level of CBX5 in a variety of BC subtype. **(F)** The mRNA expression level of CBX6 in a variety of BC subtype. **(G)** The mRNA expression level of CBX7 in a variety of BC subtype. **(H)** The mRNA expression level of CBX8 in a variety of BC subtype.

In *Xena Public Data Hubs* analysis (Figure 2), the expression of CBX2 in Basal-like and HER-2 subtypes was significantly higher than Luminal A and Luminal B subtypes of BC. However, higher mRNA expressions of CBX4 and CBX7 were detected in Luminal A and Luminal B subtypes than Basal-like and HER-2 subtypes of breast cancer. CBX1, CBX3, CBX5, CBX6 and CBX8 mRNA expression have no significant difference across various subtypes of BC.

### Elevated CBX1 expression predicted poor survival in breast cancer patients, especially in the ER positive and the subgroup treated with adjuvant chemotherapy only

**Figure 3:**
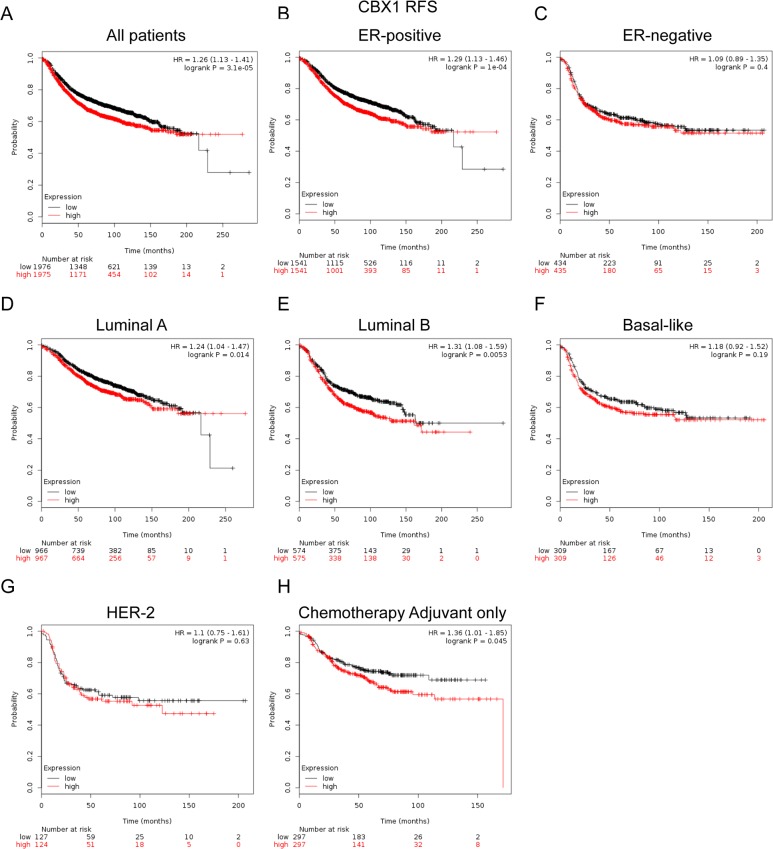
The prognostic values of CBX1 in breast cancer **(A)** Elevated CBX1 mRNA level was significantly associated with shorter RFS in all BC patients. High mRNA level of CBX1 was significantly associated with poorer RFS both in ER positive **(B)**, but not ER negative **(C)** BC patients. **(D-G)** High mRNA expression of CBX1 was significantly associated with shorter RFS in Luminal A and Luminal Bsubtype BC patients, but not in Basal-like and HER-2 subtype BC patients. **(H)** High mRNA level of CBX1 was associated with worse RFS in BC patients who have received adjuvant chemotherapy only.

We next assessed the prognostic effect of individual CBX member in patients with BC. CBX1 mRNA high expression was associated with shorter RFS in all BC patients (HR=1.26, *p*=3.1e-05) (Figure 3A). Sub-analysis indicated that CBX1 mRNA high expression was correlated to shorter RFS in BC patients with ER positive tumors (HR=1.29, *p*=1e-04) (Figure 3B), but not in ER negative tumors (HR=1.09, *p*=0.4) (Figure 3C).

Moreover, CBX1 mRNA high expression was correlated to shorter RFS in BC patients with Luminal A tumors (HR=1.24, *p*=0.014) and Luminal B tumors (HR=1.31, *p*=0.0053) (Figure 3D, 3E). However, there was no significant difference in RFS in patients either with Basal-like (HR=1.18, *p*=0.19) or HER-2 positive (HR=1.1, *p*=0.63) tumors between high and low CBX1 mRNA expression (Figure 3F, 3G).

Of noteworthy, the results demonstrated that CBX1 high expression was significantly correlated to shorter RFS in patients who have received adjuvant chemotherapy only (HR=1.36, *p*=0.045), indicating a potential role of CBX1 in contribution to chemoresistance in BC (Figure 3H). RFS sub-analysis of CBX1 chemotherapy include or exclude were provided in Supplementary Figure 8A and 8B.

### Elevated CBX2 expression predicted poor survival in breast cancer patients, especially in the ER-positive, HER-2 negative, lymph node-positive and the subgroup treated with adjuvant chemotherapy only

**Figure 4:**
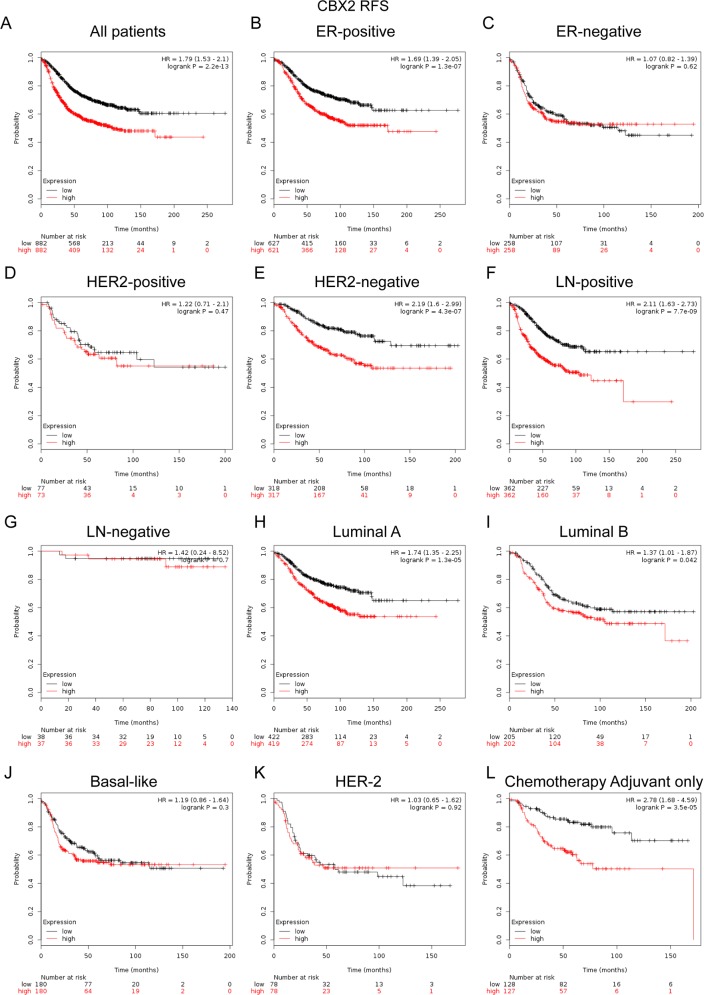
The prognostic values of CBX2 in breast cancer **(A)** Elevated CBX2 mRNA level was significantly associated with poorer RFS in all BC patients. **(B-C)** High mRNA level of CBX2 was significantly associated with shorter RFS in ER positive, but not ER negative BC patients. **(D-E)** High mRNA level of CBX2 was significantly associated with shorter RFS in HER-2 negative, but not HER-2 positive BC patients. **(F-G)** High mRNA level of CBX2 was significantly associated with shorter RFS both in LN (lymph node) positive, but not LN negative BC patients. **(H-K)** High mRNA expression of CBX2 was significantly associated with shorter RFS in Luminal A and Luminal B subtype BC patients, but not in Basal-like or HER-2 subtype BC patients. **(L)** High mRNA level of CBX2 was associated with worse RFS in BC patients who have received adjuvant chemotherapy only.

CBX2 mRNA high expression was significantly correlated with shorter RFS in all BC patients (HR=1.79, *p*=2.2e-13) (Figure 4A). CBX2 mRNA high expression was correlated to shorter RFS in BC patients with ER positive tumors (HR=1.69, *p*=1.3e-07) (Figure 4B), but not in ER negative tumors (HR=1.07, *p*=0.62) (Figure 4C). CBX2 mRNA high expression was correlated to shorter RFS in BC patients with HER-2 negative tumors (HR=2.19, *p*=4.3e-07) (Figure 4E), but not in HER-2 positive tumors (HR=1.22, *p*=0.47) (Figure 4D).

CBX2 mRNA high expression was correlated to shorter RFS in BC patients with lymph node-positive tumors (HR=2.11, *p*=7.7e-09) (Figure 4F), but not in node-negative tumors (HR=1.42, *p*=0.7) (Figure 4G). CBX2 mRNA high expression was correlated to shorter RFS in BC patients with Luminal A (HR=1.74, *p*=1.3e-05) (Figure 4H) and Luminal B tumors (HR=1.37, *p*=0.042) (Figure 4I), but not in Basal-like tumors (HR=1.19, *p*=0.3) (Figure 4J) and HER-2 subtype tumors (HR=1.03, *p*=0.92) (Figure 4K).

Notably, the results demonstrated that CBX2 high mRNA expression was significantly correlated to shorter RFS in patients who have received adjuvant chemotherapy only (HR=2.78, *p*=3.5e-05) (Figure 4L), indicating a potential role of CBX2 in contribution to chemoresistance in BC. (RFS sub-analysis of CBX2 chemotherapy include or exclude were provided in the Supplementary Figure 8C-8D)

### Elevated CBX6 expression predicted better survival in breast cancer patients, especially in the subgroup of ER-positive and HER-2 negative tumors

**Figure 5:**
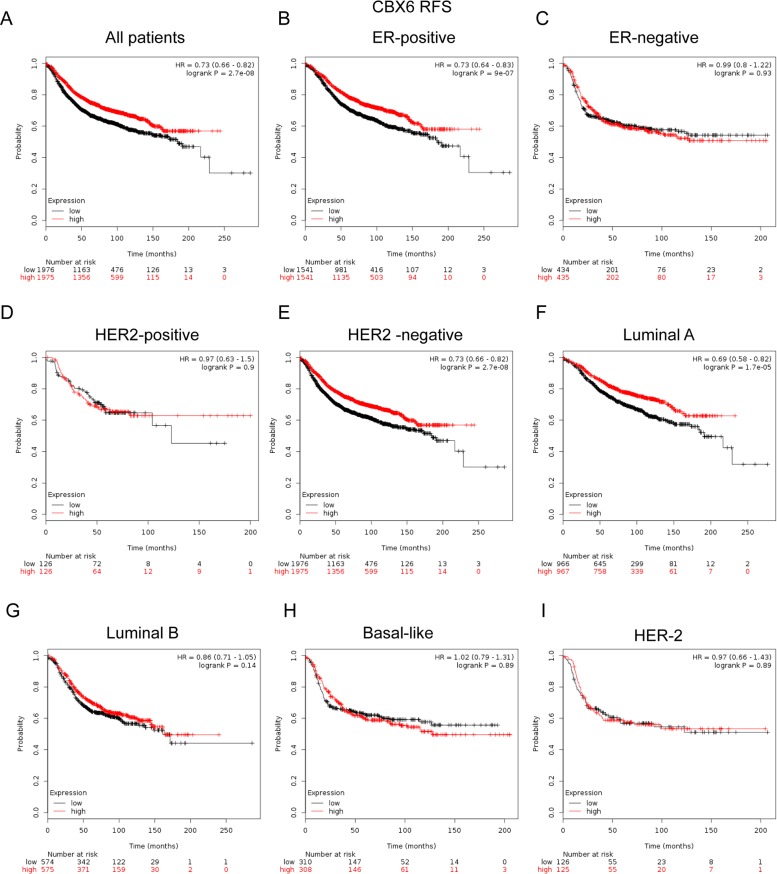
The prognostic values of CBX6 in breast cancer **(A)** High mRNA level of CBX6 was significantly associated with longer RFS in all BC patients. **(B-C)** High mRNA level of CBX6 was significantly associated with longer RFS in ER positive, but not ER negative BC patients. **(D-E)** High mRNA level of CBX6 was significantly associated with longer RFS in HER-2 negative BC patients, but not in HER-2 positive BC patients. **(F-I)** High mRNA level of CBX6 was significantly associated with longer RFS in Luminal A subtype BC patients, but not in Luminal B, Basal-like or HER-2 subtype BC patients.

In Figure 5, CBX6 high mRNA expression was significantly associated with longer RFS in all BC patients (HR=0.73, *p*=2.7e-08) (Figure 5A). In particular, sub-analysis revealed that high mRNA expression of CBX6 was significantly associated with better survival in ER positive (HR=0.73, *p*=9e-07) (Figure 5B), HER-2 negative tumors (HR=0.73, *p*=2.7e-08) (Figure 5E), and Luminal A subtype patients (HR=0.69, *p*=1.7e-5) (Figure 5F), but not in HER-2 positive (HR=0.97, *p*=0.9) (Figure 5D), Luminal B subtype patients (HR=0.86, *p*=0.14) (Figure 5G), Basal-like subtypes BC patients (HR=1.02, *p*=0.89) or HER-2 subtype (HR=0.97, *p*=0.9) (Figure 5H, 5I).

### Elevated CBX7 expression predicted better survival in breast cancer patients, especially in the subgroup treated with tamoxifen only and adjuvant chemotherapy only

**Figure 6:**
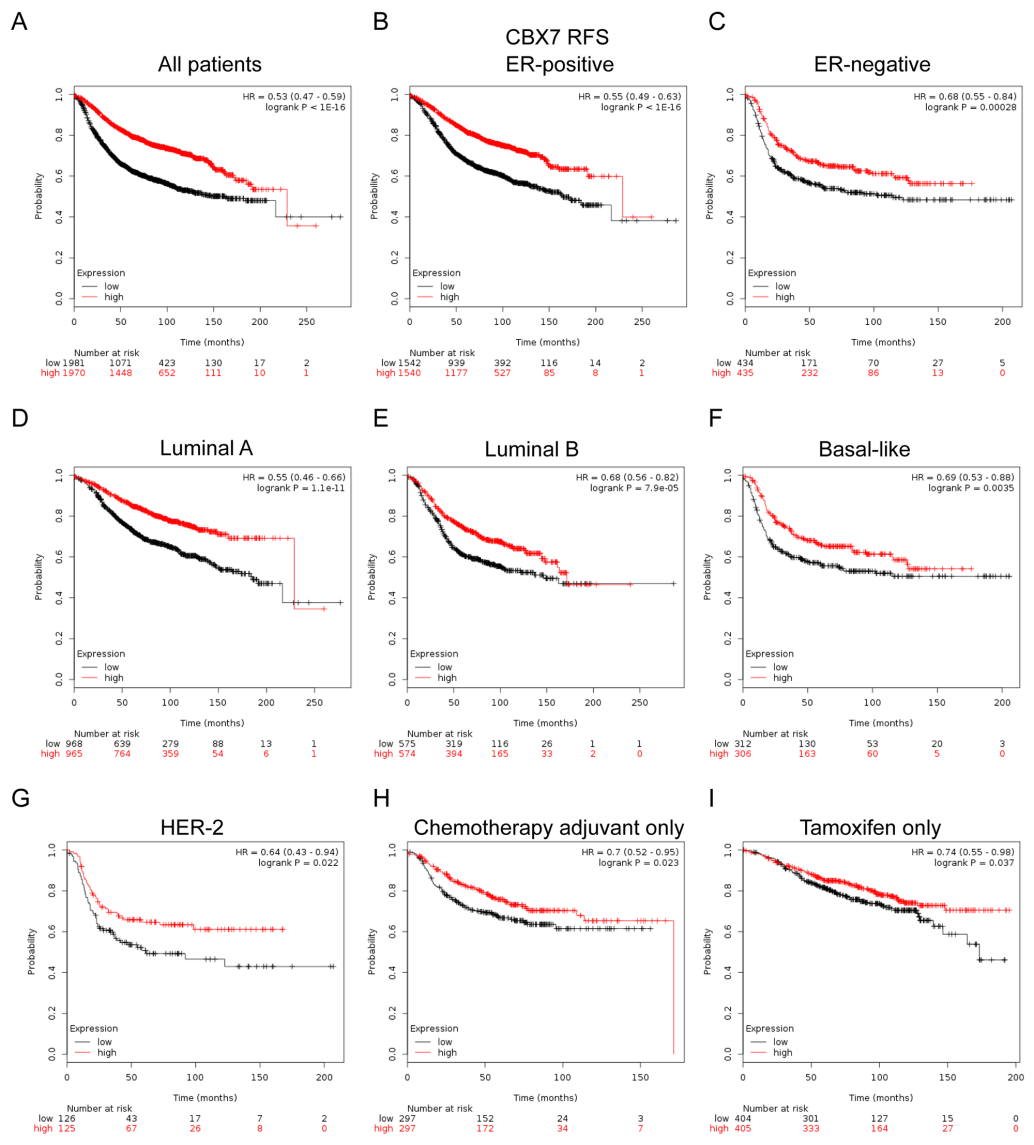
The prognostic values of CBX7 in breast cancer **(A)** High mRNA level of CBX7 was significantly associated with longer RFS in all BC patients. **(B-C)** High mRNA level of CBX7 was significantly associated with longer RFS in both ER positive and ER negative BC patients. **(D-G)** High mRNA level of CBX7 was significantly associated with better RFS in Luminal A, Luminal B, Basal-like and HER-2 subtype BC patients. **(H, I)** High mRNA level of CBX7 was associated with longer RFS in BC patients who have received adjuvant chemotherapy only or tamoxifen only subgroup.

In Figure 6, CBX7 high mRNA expression was significantly associated with better prognosis in all BC patients (HR=0.53, *p*<1e-16) (Figure 6A). In particular, sub-analysis revealed that high mRNA expression of CBX7 was significantly associated with better survival in both ER positive (HR=0.55, *p*<1e-16) and ER negative (HR=0.68, *p*=0.00028) patients (Figure 6B, 6C), as well as those with Luminal A (HR=0.55, *p*=1.1e-11) and Luminal B (HR=0.68, *p*=7.9e-05) tumors (Figure 6D, 6E). Similar trend was also found in patients with Basal-like (HR=0.69, *p*=0.0035) (Figure 6F) and HER-2 subtype tumor (HR=0.64, *p*=0.022) (Figure 6G).

Of noteworthy, the results demonstrated that CBX7 high mRNA expression was significantly correlated to longer RFS in patients who treated with tamoxifen only (HR=0.74, *p*=0.037) (Figure 6I), indicating a potential role of CBX7 in contribution to tamoxifen sensitivity in breast cancer. RFS sub-analysis of CBX7 endocrine-therapy include or exclude were provided in Supplementary Figure 8E and 8F. The results also demonstrated that CBX7 high mRNA expression was significantly correlated to longer RFS in patients who have received adjuvant chemotherapy only (HR=0.7, *p*=0.023) (Figure 6H), indicating a potential role of CBX7 in contribution to chemosensitivity in breast cancer.

## DISCUSSION

Breast cancer (BC) is virtually a heterogeneous disease that comprising a variety of subtypes associated with distinct biological and clinical features [21, 22]. Latest technologies have unraveled the molecular underpinning of several characteristics of breast cancer, including histological grade and metastatic propensity, and have led to the identification of prognostic and predictive gene expression signatures [23, 24].

Apart from abnormality in transcriptome, disorder in epigenetic regulation also plays significant role in the tumorigenesis and development of BC [1, 25]. Chromobox (CBX) family proteins are canonical components in PRC1(polycombrepressive complexes 1), which is one of the best characterized polycomb group (PcG) complexes, with epigenetic regulatory function of conducting transcriptional repression of target genes via modifying the chromatin [7, 26].

Previously, several groups have shown that aberrant CBX member expression were observed in certain cancers tissues [27–29]. CBX1 levels were prominent in prostate cancer [30]. CBX4 was elevated in Hepatocellular carcinoma [31] whereas CBX6 was decreased in glioblastoma [32]. CBX7 was upregulated in prostate cancer [33] and gastric cancer [34] while declined in thyroid cancer [35], lung cancer and colon cancer [36, 37]. However, litter is understood in the expression of individual CBX member in breast cancer.

Results from our analysis suggested that almost all CBX family members were distinctively high-expressed in BC comparing to normal controls, implying their unique roles in BC. Even though no data of CBX1 in the dataset, a study by Young-Ho Lee and colleagues reported that CBX was heterogeneously distributed in BC samples and high expression of CBX1 had a positive correlation to Ki-67 and associated with poorly differentiated breast tumors and with a significantly poor prognosis [38].

Our finding demonstrated that, through using *Xena* database analysis, the expression of CBX2 in Basal-like and HER-2 subtypes was significantly higher than Luminal subtypes breast cancer. This was consistent with the report by Chen and colleagues that protein expression of CBX2 was significantly higher in tumor tissues than adjacent normal tissues, and similarly, CBX2 was found to be significantly associated with positive HER-2 status [39]. Moreover, higher mRNA expressions of CBX4 and CBX7 were detected in Luminal subtypes than Basal-like subtypes of breast cancer. The disparate enrichment of CBX proteins in various subtypes of breast cancer may be one of the underlying mechanisms pertinent to different biological properties, as well as diverse prognosis.

In the current study, it was found that CBX1, CBX2 and CBX3 mRNA high expression was correlated to worsen RFS for all BC patients (Figure 3, 4; Supplementary Figure 4). CBX4, CBX5, CBX6 and CBX7 mRNA high expression was correlated to better RFS for all BC patients (Figure 5, 6; Supplementary Figure 5, 6). No significantly difference was found between the group of CBX8 high expression and low expression in RFS for all BC patients (Supplementary Figure 7). RFS sub-analysis of CBX3, CBX4, CBX5, CBX8 were also provided in Supplementary Figure 4-7. Our study further demonstrated that high CBX1 expression predicts poor survival in BC patients, especially in those with ER-positive tumors. These results partly agree with report by Young-Ho Lee and colleagues that high CBX1 expression group displayed more aggressive types of breast cancers, including Basal-like and Luminal B subtypes, and associated with shorter disease-free survival (DFS) and overall survival (OS) [38]. However, more evidences remain needed to validate different expression profiles of CBX1 in various molecular subtypes of BC.

It was suggested in the study that high mRNA expression of CBX2 predicts poor survival in BC patients. In line with several studies reporting that CBX2 was a potential drug target in human cancers. In a human study, the depletion of CBX2 resulted in decreased cell proliferation and increasing levels of apoptosis, emphasizing the requirement of CBX2 in the maintenance of hematopoietic stem cell [40]. CBX2 was found to be phosphorylated in cell lines that proliferate rapidly in culture, whereas it was not phosphorylated in most tissues examined [41]. CBX2 protein expression levels were inversely associated with prognosis in breast cancer patients. CBX2 expression was associated with clinical features, including positive lymph node metastasis status, large tumor size and HER-2 positive status [14]. CBX2 upregulation and amplification significantly correlated with metastatic progression and lower overall survival in many cancer types, particularly those of the breast [39].

Moreover, in subgroup analysis, we found that elevated CBX1 and CBX2 expression were correlated to worse prognosis in the patients treated with adjuvant chemotherapy only, which indicated that CBX1 and CBX2 might play pivotal roles in chemoresistance. Young-Ho and colleagues found that HP1β (CBX1) depleted BC cells were hypersensitive to PARP inhibitor, compromised HP1βabundance may serve as a useful predictive marker for chemotherapy [38]. Chen and colleagues revealed that OS time of BC patients treated with Taxol was significantly lower than that of patients who did not receive Taxol treatment in the high CBX2 expression group. However, no significant difference in OS time was identified between patients treated with or without Taxol in the low CBX2 expression group [14]. Therefore, CBX2 might be a novel biomarker for the selection of appropriate chemotherapy regimens for BC patients.

Although study showed that induced CBX6 overexpression inhibited glioblastoma cell proliferation [32], which implying CBX6 to be a tumor suppressor in certain cancer. However, few researches covered the prognostic value of CBX6 in BC. In our study, we first reported that elevated CBX6 expression predicted better survival in BC patients, specifically in the subgroup with ER positive and HER-2 negative tumors. Therefore, it is extrapolated that CBX6 might be an independent prognostic factor in BC.

Most interestingly, our study suggested that elevated CBX7 expression predicted better survival in BC patients, and was correlated with poor prognosis in patients treated with tamoxifen only and adjuvant chemotherapy only. CBX7 is the most characterized CBX protein in cancer-associated studies which plays a critical role in cancer progression [12]. Also, Ni, S *et al.* reported that CBX7 inhibited cell proliferation, migration, and invasion through the inhibition of PTEN/AKT signaling in pancreatic cancer [42]. Studies showed that loss of the CBX7 expression correlated with a highly malignant phenotype in thyroid cancer [35], or a more aggressive biological behavior in pancreatic cancer [13]. Multivariate analysis showed that combined EZH2/CBX7 status was an independent prognostic factor [43].

Specially, it has been reported that CBX7 inhibited breast tumorigenicity through DKK-1-mediated suppression of the Wnt signaling pathway, suggesting that CBX7 was a critical tumor suppressor in human BC [44]. In subgroup analysis, we found that high expression of CBX7 was significantly correlated to longer RFS in patients who have received adjuvant chemotherapy only, indicating a potential role of CBX7 in contribution to chemosensitivity in BC. Also, Cacciola and colleagues revealed that restoration of CBX7 expression increased the susceptibility of human lung carcinoma cells to irinotecan treatment [45]. Therefore, enhancement of CBX7 expression might be a potential measure for improving treatment response to chemotherapy.

In summary, CBX1, CBX2 and CBX3 mRNA high expression was associated with worsen RFS for all BC patients. CBX4, CBX5, CBX6 and CBX7 mRNA high expression was correlated to better RFS for all BC patients. CBX1 and CBX2 might be important predictor of chemoresistance in breast tumors. High level of CBX7 in breast tumors might portend a better response when treated with tamoxifen or chemotherapeutical agents. CBX1, CBX2 and CBX7 could be potential targets of individualized therapy for BC patients. These results will help to better understand the complexity and heterogeneity in the molecular biology of BC, as well as to develop strategy to more accurately tailor treatment and predict survival outcome for patients with BC.

## MATERIALS AND METHODS

### *CCLE* analysis

The mRNA levels of CBX members in a series of cancers were analyzed by *CCLE* database (https://portals.broadinstitute.org/ccle/home), which is an online encyclopedia of a compilation of gene expression, chromosomal copy number and massively parallel sequencing data from 947 human cancer cell lines, to facilitate the identification of genetic, lineage, and predictors of drug sensitivity, as described in our previous study [46].

### *ONCOMINE* analysis

The mRNA levels of distinct CBX family members in diverse cancer types were determined through analysis in *ONCOMINE* database (www.oncomine.org), which is a publicly accessible online database with cancer microarray information to facilitate discovery from genome-wide expression analyses.

In this study, a *p*-value was calculated through students’*t*-test for comparison between datasets derived from cancer specimens and normal controls. The fold change was defined as 2 and *p* value was set up at 0.01. Significant correlations can be found in a series of BC researches, as displayed in typical figures.

### *Xena* public data hubs

UCSC Xena browser (http://xena.ucsc.edu/) was used to obtain The Cancer Genome Atlas (TCGA) breast cancer data and to evaluate CBX family members’ mRNA expression in different subtypes of human breast cancer.

### The Kaplan-Meier plotter survival analysis

Prognostic values of featured CBX members that found specifically high expressed in BC samples were subsequently evaluated by showing the relapse-free survival (RFS), which was based on the analysis in the Kaplan-Meier plotter (www.kmplot.com). The log-rank P was calculated accordingly, as shown on the webpage. [47]

## SUPPLEMENTARY MATERIALS FIGURES


