# Supplementary material for: Prognostic values of distinct CBX family members in breast cancer
Source: Oncotarget. 2017 Sep 28;8(54):92375–87. doi: 10.18632/oncotarget.21325 (PMC5696189; doi:10.18632/oncotarget.21325)
Supplement: Supplementary file 1 [file oncotarget-08-92375-s001.pdf]

Prognostic values of distinct CBX family members in breast cancer

SUPPLEMENTARY MATERIALS

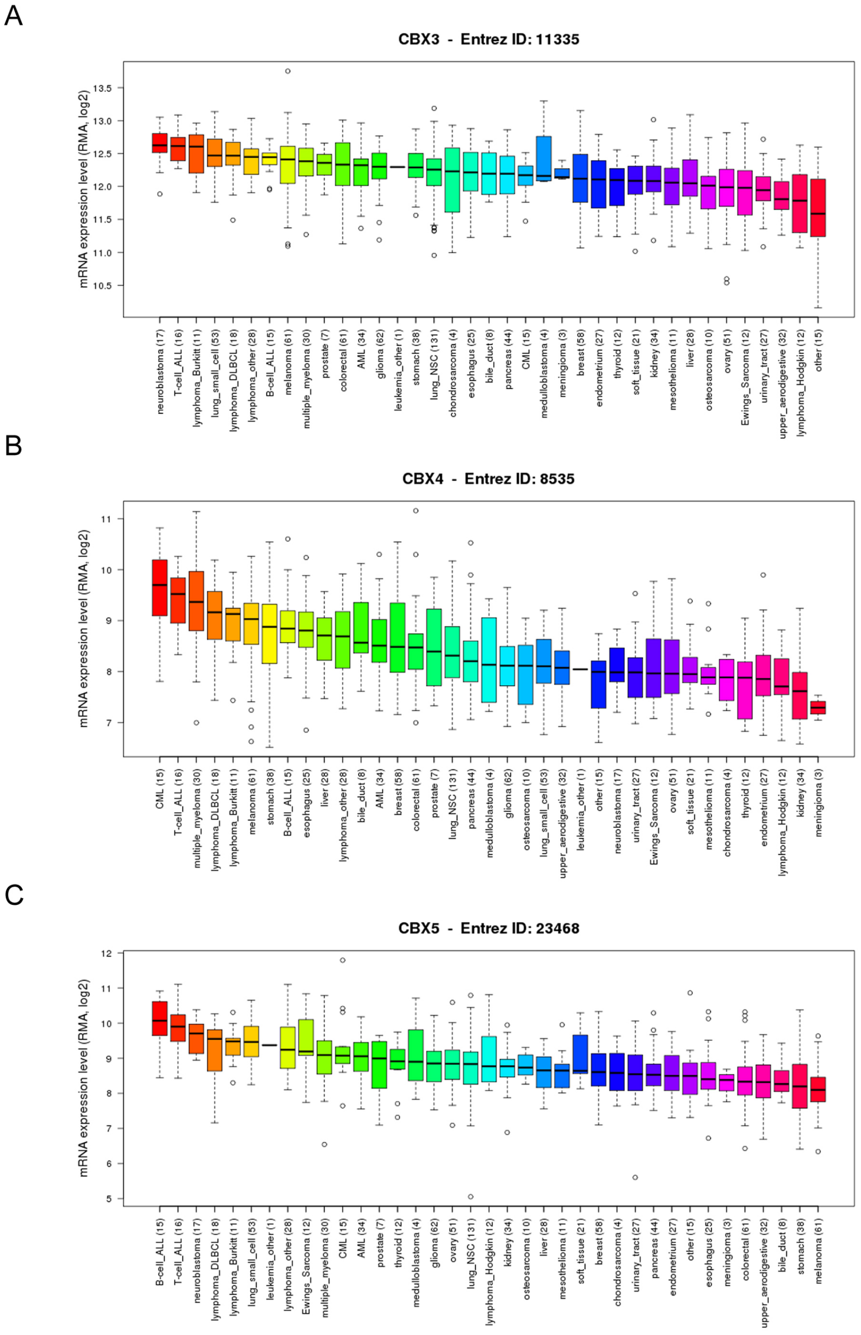

Supplementary Figure 1: CBX family members CBX3, CBX4, CBX5 are distinctively overexpressed in breast cancer cell lines from CCLE analysis. **(A)** The mRNA expression level of CBX3 in a variety of cancer cell line. **(B)** The mRNA expression level of CBX4 in a variety of cancer cell line. **(C)** The mRNA expression level of CBX5 in a variety of cancer cell line.

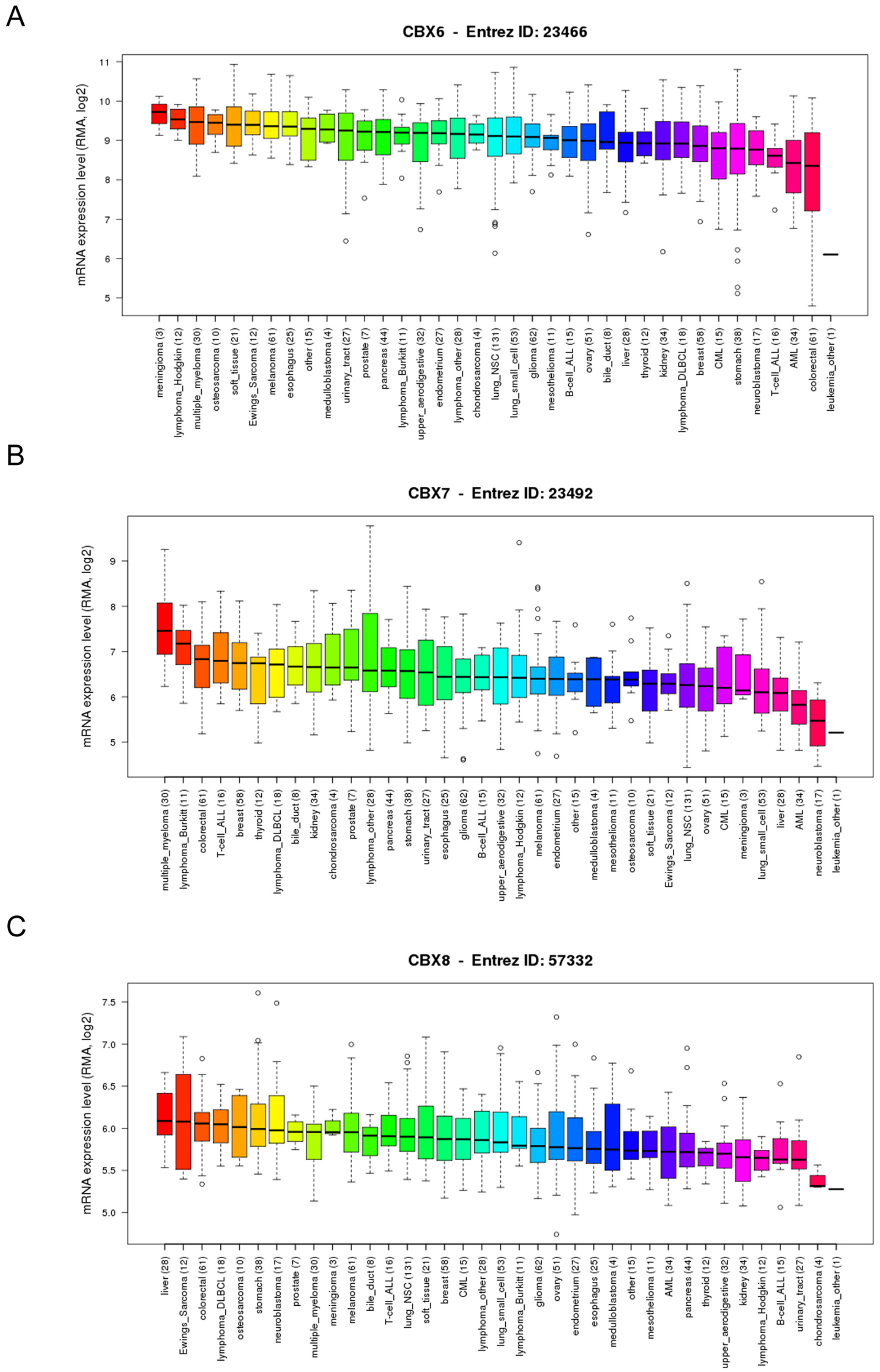

Supplementary Figure 2: CBX family members CBX6, CBX7, CBX8 are distinctively overexpressed in breast cancer cell lines from CCLE analysis. **(A)** The mRNA expression level of CBX6 in a variety of cancer cell line. **(B)** The mRNA expression level of CBX7 in a variety of cancer cell line. **(C)** The mRNA expression level of CBX8 in a variety of cancer cell line.

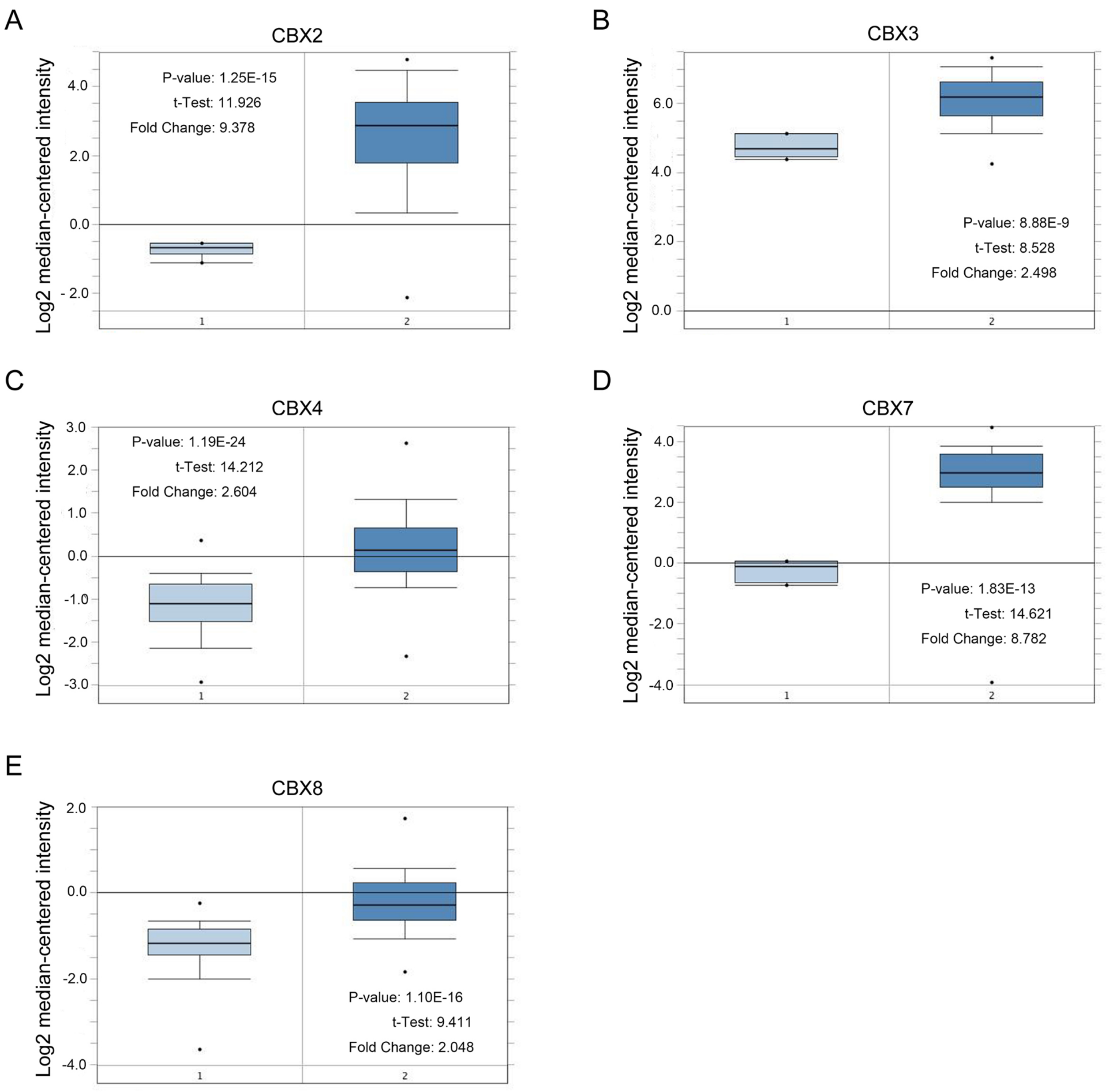

Supplementary Figure 3: CBX family analysis in breast cancer (ONCOMINE database). Box plots derived from gene expression data in ONCOMINE comparing expression of a specific CBX family member in normal and BC tissue. The p value was set up at 0.01 and fold change was defined as 2. **(A)** Comparison of CBX2 mRNA expression. **(B)** Comparison of CBX3 mRNA expression. **(C)** Comparison of CBX4 mRNA expression. **(D)** Comparison of CBX7 mRNA expression. **(E)** Comparison of CBX8 mRNA expression.

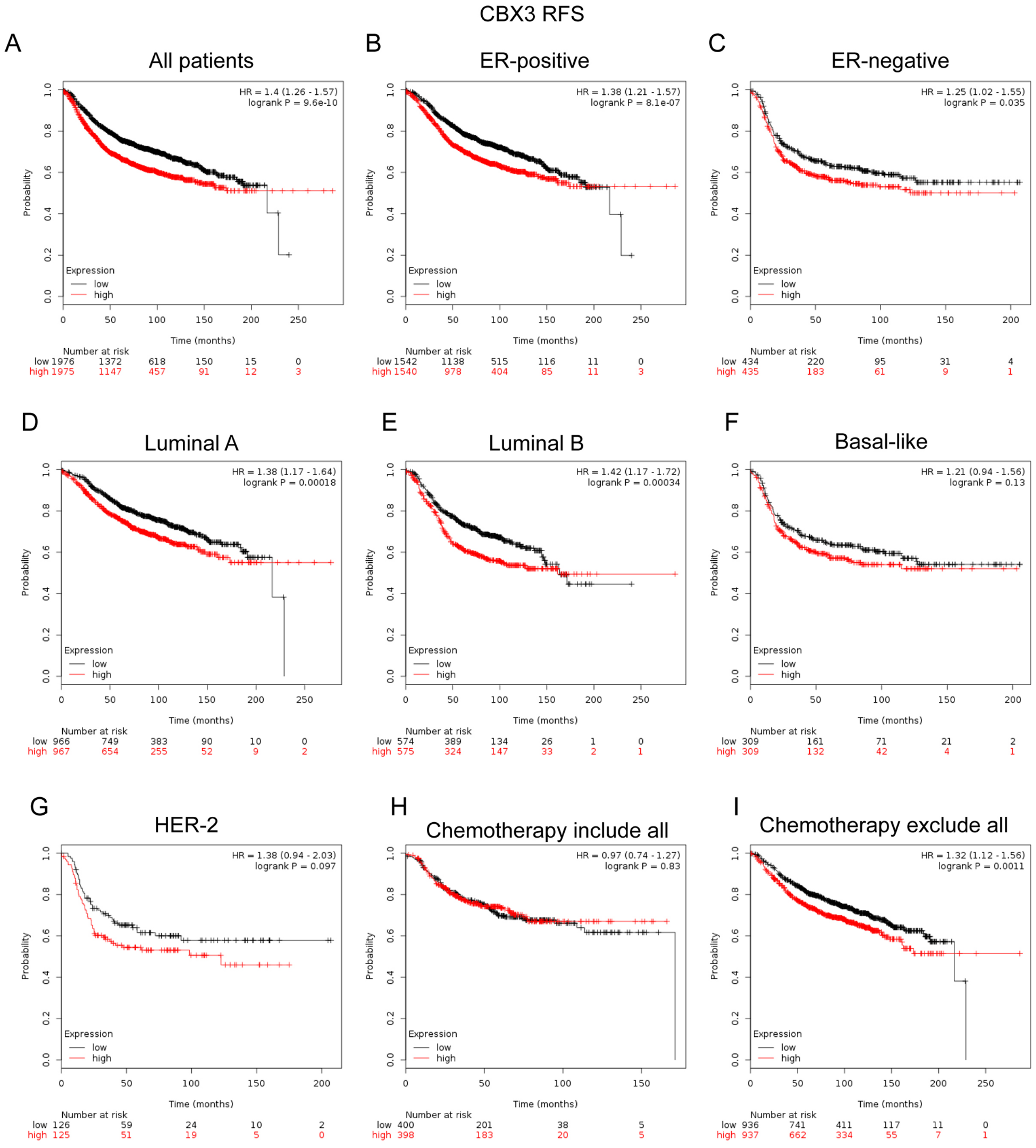

Supplementary Figure 4: The prognostic values of CBX3 mRNA in breast cancer. (A) Low mRNA level of CBX3 was associated with longer RFS in all BC patients. **(B, C)** Low mRNA level of CBX3 was associated with longer RFS both in ER+ and ER- BC patients. **(D-G)** Low mRNA level of CBX3 was associated with longer RFS in luminal A, Luminal B subtype but not in HER-2, Basal-like BC patients. **(H, I)** Low mRNA level of CBX3 was associated with longer RFS in BC patients without adjuvant chemotherapy, but not in BC patients within adjuvant chemotherapy.

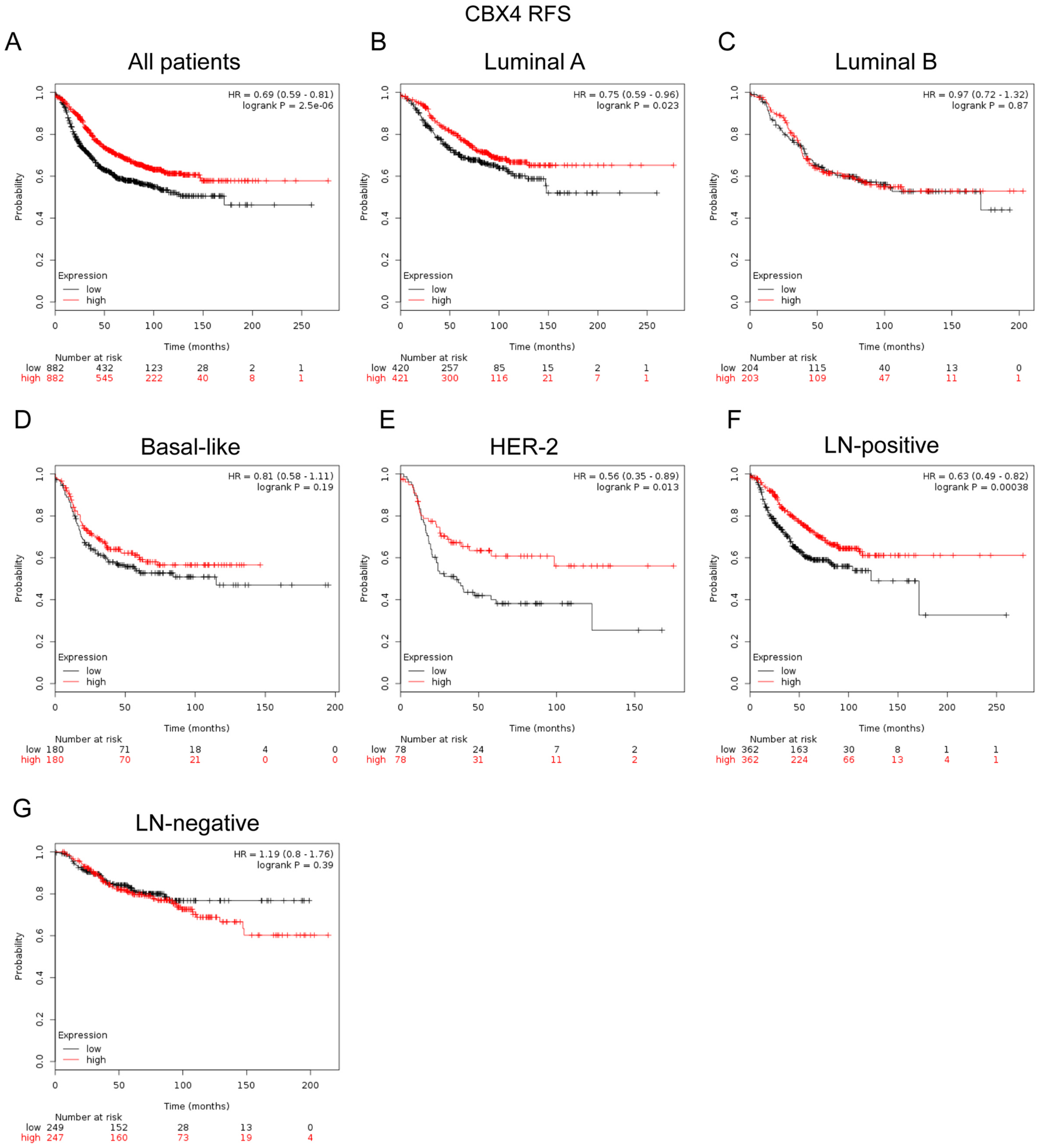

Supplementary Figure 5: The prognostic values of CBX4 mRNA in breast cancer. (A) High mRNA level of CBX4 was associated with longer RFS in all BC patients. **(B-E)** High mRNA level of CBX4 was associated with longer RFS in luminal A, HER-2 subtype but not in Luminal B, Basal-like BC patients. **(F, G)** High mRNA level of CBX4 was associated with longer RFS in LN(+) BC patients, but not in LN(-) BC patients.

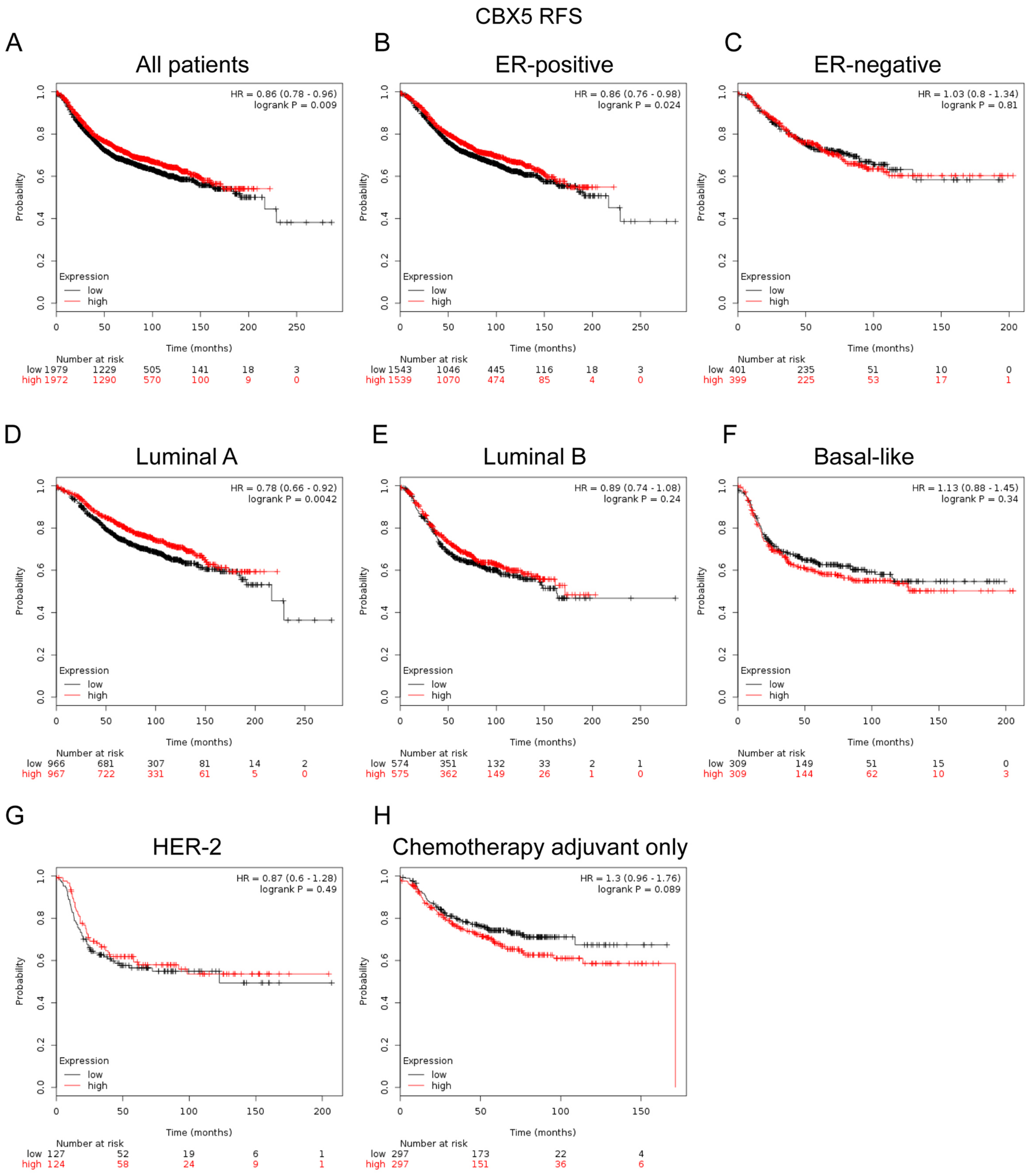

Supplementary Figure 6: The prognostic values of CBX5 mRNA in breast cancer. **(A)** High mRNA level of CBX5 was associated with longer RFS in all BC patients. **(B, C)** High mRNA level of CBX5 was associated with longer RFS in ER+, but not ER- BC patients. **(D-G)** High mRNA level of CBX5 was associated with longer RFS in luminal A subtype BC patients, but not in luminal B, HER-2, Basal-like subtype BC patients. **(H)** Low mRNA level of CBX5 was associated with longer RFS in BC patients received chemotherapy only.

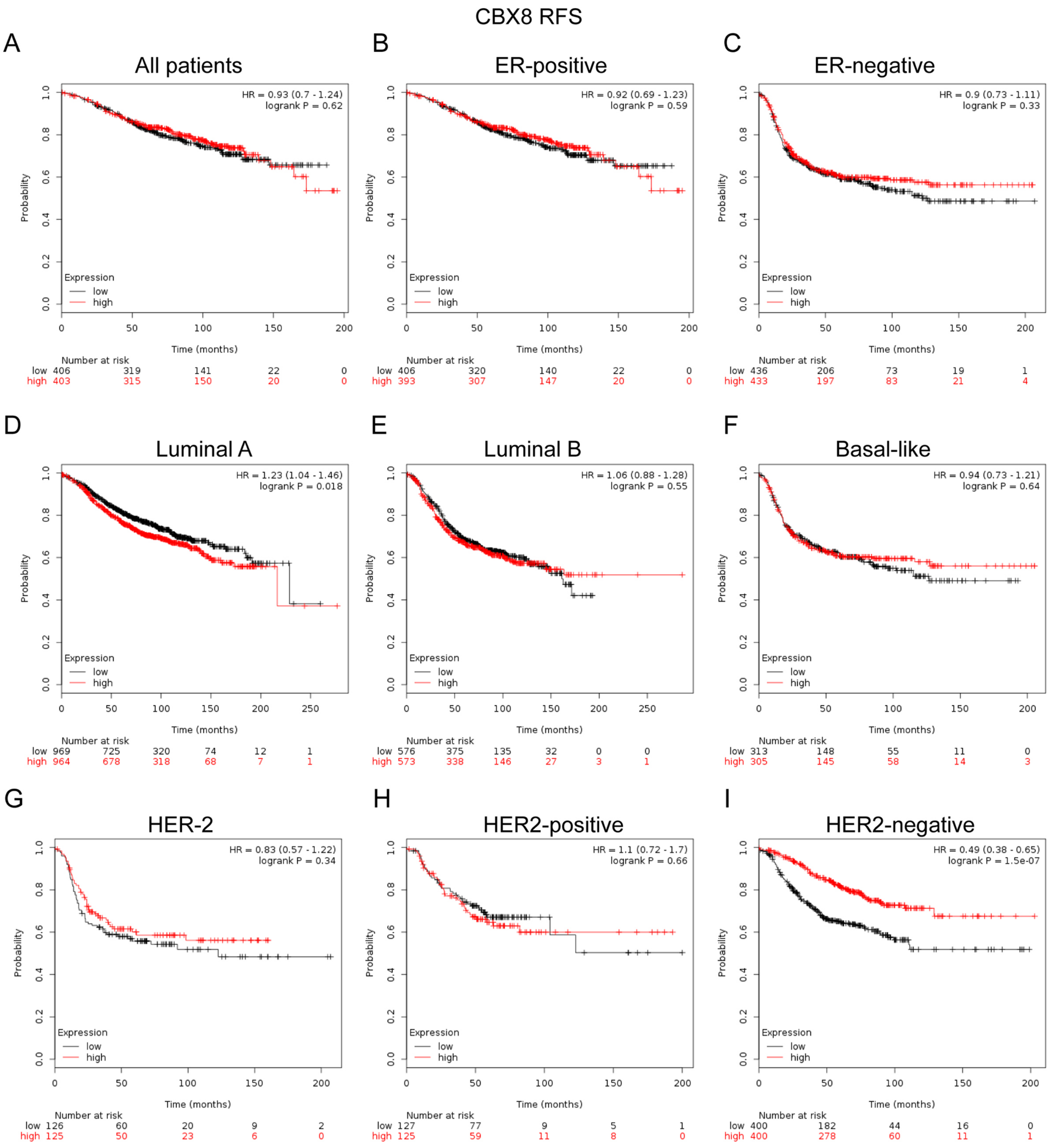

Supplementary Figure 7: The prognostic values of CBX8 mRNA in breast cancer. (A-C) There was no significant difference in RFS between high and low level of CBX8 expression, both in ER+ or ER- BC patients. **(B)** High mRNA level of GATA3 was associated with longer RFS both in ER+, but not ER- BC patients. **(D-G)** Low mRNA level of CBX8 was associated with longer RFS in luminal A subtype BC patients, but not in luminal B, HER-2, Basal-like subtype BC patients. **(H, I)** High mRNA level of CBX8 was associated with longer RFS in HER-2(–), but not HER-2(+)BC patients.

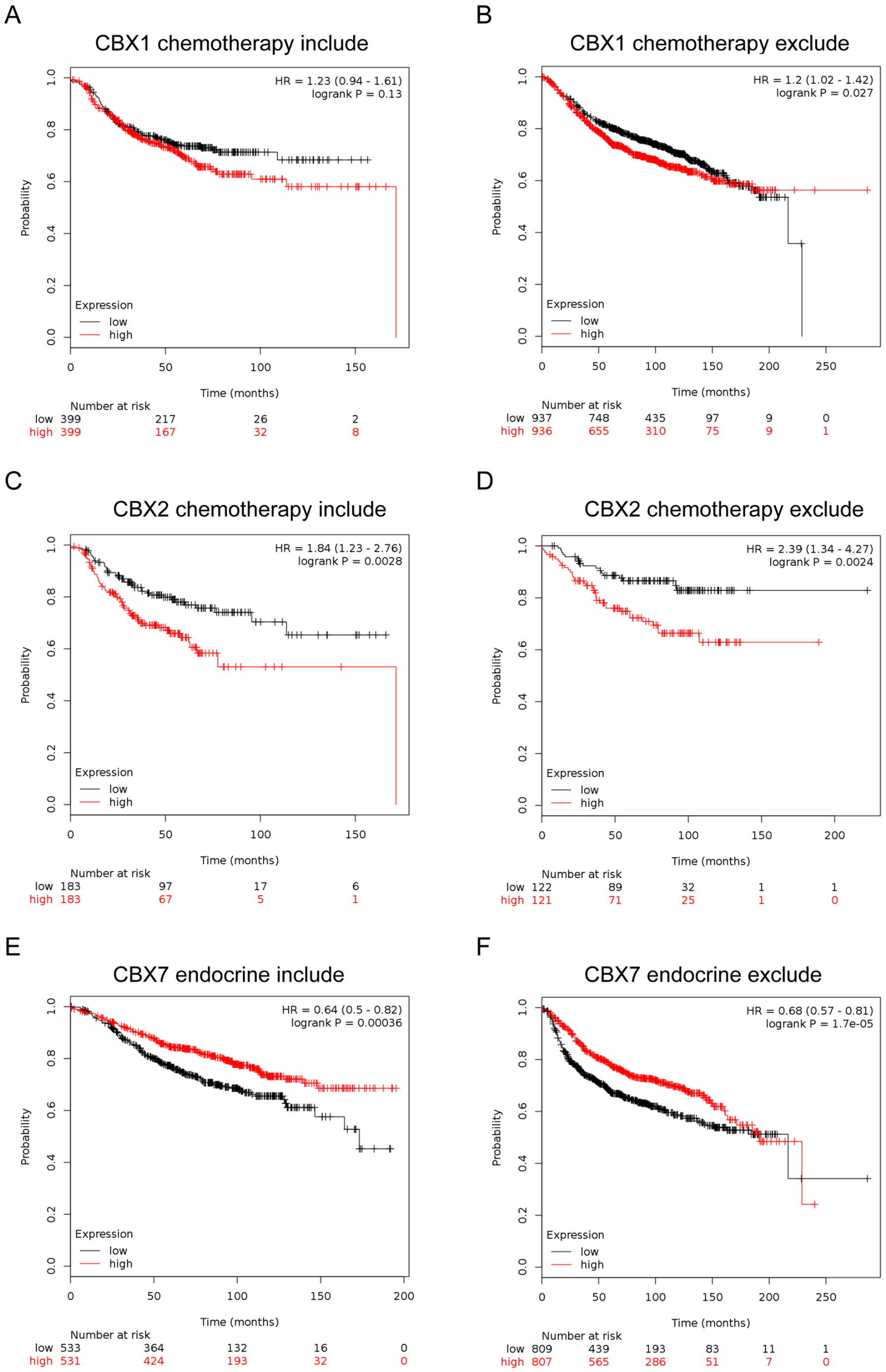

Supplementary Figure 8: The prognostic values of CBX members in the subgroup of chemotherapy and endocrine therapy BC patients. (A, B) High mRNA level of CBX1 was significantly associated with shorter RFS in the subgroup of chemotherapy exclude, but not in chemotherapy include BC patients. **(C, D)** Elevated mRNA level of CBX2 was associated with shorter RFS in the subgroup of chemotherapy include and chemotherapy include BC patients. **(E, F)** High mRNA level of CBX7 was associated with longer RFS in BC patients who have received endocrine include or t endocrine exclude subgroup.
